# The role of isoflavones in augmenting the effects of radiotherapy

**DOI:** 10.3389/fonc.2022.800562

**Published:** 2023-03-01

**Authors:** Alesia Ivashkevich

**Affiliations:** ^1^ Faculty of Engineering and Information Sciences, University of Wollongong, Wollongong, NSW, Australia; ^2^ Noxopharm, Gordon, NSW, Australia

**Keywords:** Idronoxil (Veyonda/phenoxodiol), ENOX2, topoisomerase, radiation therapy, apoptosis, prostate cancer, ER receptor, isoflavones

## Abstract

Cancer is one of the major health problems and the second cause of death worldwide behind heart disease. The traditional soy diet containing isoflavones, consumed by the Asian population in China and Japan has been identified as a protective factor from hormone-related cancers. Over the years the research focus has shifted from emphasizing the preventive effect of isoflavones from cancer initiation and promotion to their efficacy against established tumors along with chemo- and radiopotentiating effects. Studies performed in mouse models and results of clinical trials emphasize that genistein or a mixture of isoflavones, containing in traditional soy diet, could be utilized to both potentiate the response of cancer cells to radiotherapy and reduce radiation-induced toxicity in normal tissues. Currently ongoing clinical research explores a potential of another significant isoflavone, idronoxil, also known as phenoxodiol, as radiation enhancing agent. In the light of the recent clinical findings, this article reviews the accumulated evidence which support the clinically desirable interactions of soy isoflavones with radiation therapy resulting in improved tumor treatment. This review discusses important aspects of the development of isoflavones as anticancer agents, and mechanisms potentially relevant to their activity in combination with radiation therapy of cancer. It gives a critical overview of studies characterizing isoflavone targets such as topoisomerases, ENOX2/PMET, tyrosine kinases and ER receptor signaling, and cellular effects on the cell cycle, DNA damage, cell death, and immune responses.

## Introduction

Cancer represents a major health problem worldwide and its incidence is predicted to increase in the future (1). Radiation therapy is a highly cost-effective cancer treatment received by approximately 50% of all cancer patients as a single modality, or in combination with chemotherapy (2).

The occurrence and mortality risk of hormone-related cancers including prostate, breast, endometrium, and ovarian cancers is much higher in the United States and Europe compared to Asian countries such as Japan and China (3, 4). Epidemiologic studies have demonstrated that the observed differences are attributed to the traditional diet rich in soy products (3, 5) alongside of a broad spectrum of dietary and lifestyle factors (6, 7). It has been estimated that in Asia soy consumption constitutes around 15-50 mg/day, and in western countries 2 mg/day (8, 9). The isoflavones genistein, daidzein, and glycitein - polyphenolic plant-derived compounds - are mainly found in soybeans and these compounds have been shown to be the principal biologically active anticancer molecules (10, 11). They have been described as non-steroid phytoestrogens that have both estrogenic and antiestrogenic activity. In plants, isoflavones are mostly present in the inactive form as glycosides (genistin and daidzin). Following consumption, isoflavone glycosides are hydrolyzed in intestines by bacterial beta-glucosidases and converted to the corresponding bioactive aglycones such as genistein and daidzein (12). They are being absorbed from the intestine to blood and conjugated mainly in the liver to glucuronides which are excreted in the urine. Genistein and daidzein are the major isoflavones detected in the blood and urine of humans (13). Human dietary studies demonstrate that -90% of the total amount of genistein exists in either the glucoronidated or sulphated form (14), while only 10% exists in the free nonconjugated from (15), which have different biologic activity (16, 17). Marked differences between humans, rats, and mice in the profile of major metabolites following phase II metabolism were observed (18).

Anticancer activity of isoflavones in hormone-dependent (prostate, breast, ovary, and endometrium) and hormone-independent cancers (lung, melanoma) has been extensively studied experimentally in *in vivo* and *in vitro* cancer models (19–21). Dietary supplementation with soy significantly prevented spontaneous and chemically induced prostate carcinogenesis in rat models (22–25) and inhibited growth and metastasis of androgen-sensitive human prostate cancers (PC) in an orthotropic mouse model (26). Growth of PC3 and LNCaP cells have been reported to be inhibited independently of their p53 and AR status (24, 27, 28). In women, epidemiological data indicate that dietary isoflavone consumption is inversely related with the risk of breast (20, 29, 30), endometrial (21), ovarian (31), and cervical cancers (32). Furthermore, chemopreventive properties of isoflavones were explored in rat (33–36) and mouse models of mammary (37, 38) and endometrial (39) cancers.

*In vitro*, genistein inhibited proliferation of hormone independent leukaemia (40), lymphoma (41, 42), melanoma (19), lung (43), pancreatic (42, 44), gastric (44), intestinal, hepatic (45), urinary (46), and head and neck cancer cells (47).

Although anticancer properties of genistein, an active and predominant dietary isoflavone, have been addressed by large number of studies, more recent research focuses on anticancer activity of Idronoxil, being currently developed under the Veyonda^®^ trademark. Idronoxil is a synthetic isoflavone metabolite that is a natural intermediate (dehydroequol, 7,40-dihydroxyisoflav-2-ene) in the metabolism of daidzein to equol (48). Analogously to genistein, however more potently, it exhibits anticancer activity in several tumor types as was demonstrated in head and neck (49), ovarian (50), renal (51) cell lines, and prevents carcinogenesis (52). Several clinical trials have addressed Idronoxil activity as an agent potentiating radiation therapy.

## Mechanisms of soy isoflavones as anticancer agents

**Figure 1:**
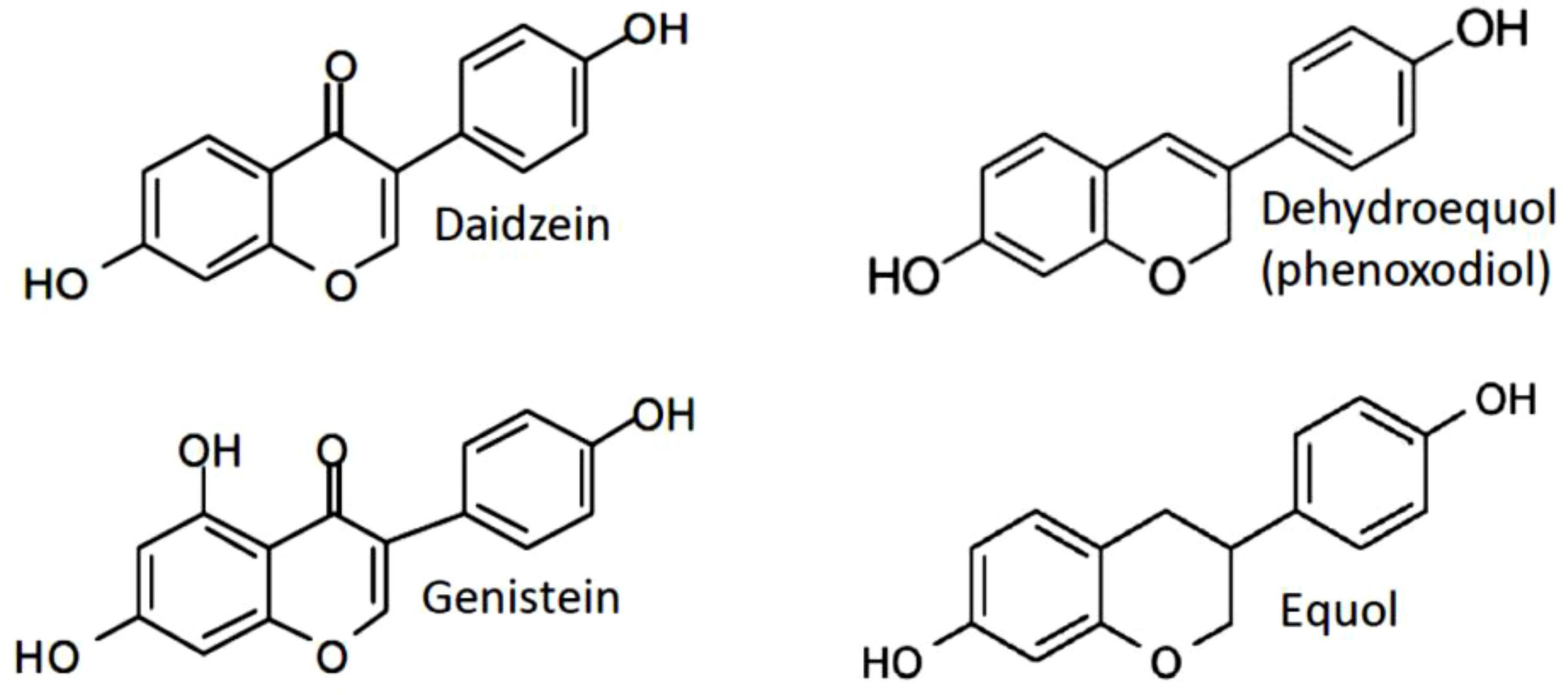
Structures of the mainly studied isoflavones daidzein, genistein, phenoxodiol (idronoxil) and equol compared to estradiol-17 β.

Scientific studies have mainly addressed the mechanisms of antitumor effectiveness of genistein, daidzein and its metabolites phenoxodiol (idronoxil) and equol. All isoflavones display multifaceted cellular responses resulting from their ability to target several essential cellular processes. Interestingly, despite the same origin and some degree of structural similarity, genistein and daidzein appear to be significantly different in their mechanism of action. Equol, the final product of metabolic conversion of daidzein, has also been found to demonstrate differential activities when compared to daidzein. The chemical structure of equol is substantially similar to its precursor phenoxodiol (**Figure 1**). However, no detailed comparative study of its properties to those of phenoxodiol has been performed to date.

### Non-steroidal action of isoflavones on ER receptor linked to antitumor effects

Genistein, daidzein, and the daidzein metabolite equol show structural similarity to estradiol-17 β (E2), and exhibit estrogenic properties. It has been demonstrated that soy phytoestrogens and their metabolites bind to and activate both estrogen receptor (ER) α and β (53, 54). They have been described to exhibit very diverse pro- and anti-estrogenic effects on ER related cell function in various cell types including immune cells (55–58). Comparing to estradiol, these compounds possess relatively limited estrogenic potency. For example, equol shows 10^-3^-10^-5^ times the activity of 17β-estradiol (59, 60). However, their high levels in humans under certain nutritional conditions or pharmacological delivery could induce significant biological effects. The following sequence of the efficacy of activation of ERα has established (53), with the EC(50):  17β-estradiol (0.03 μM) > equol (3.5 μM) > genistein (15 μM) > daidzein (>300 μM), and for ERβ 17β-estradiol (0.01 μM) > genistein (0.03 μM) > daidzein (0.35 μM) > equol (0.4 μM). The results of another study (61) suggested that in cells with a predominance of ER-α genistein acts as an agonist to ER-α, and in cells with ER-β alone genistein most likely acts as an anti-estrogen.

Action of isoflavones on ER receptors could result in a direct antitumor activity as ER-β appears to play an antiproliferative role in healthy prostate (62–64). Furthermore, ER-β was also found to inhibit epithelial proliferation and diminish breast cancer risk and progression (65, 66).

### Topoisomerase inhibition, DNA damage, and clastogenic potential of isoflavones

Early studies have established that genistein is a potent inhibitor of both topoisomerases I and II (67). Subsequently it has been ascribed properties of non-intercalating topoisomerase poison (67–69). It was found to have an effect on the cleavage activity of DNA topoisomerase II, which resulted in the induction of protein-linked DNA strand breaks (70). Topoisomerase II-mediated DNA breakage was demonstrated in colorectal cancer cells in response to genistein (70). Another study found a significant induction of double strand breaks as detected with γH2AX assay in A549 lung cancer cells at 24 and 48 hrs treatment with 30 µM of soy isoflavones. 40-50% of the mixture of soy isoflavones [SIF, extract from soybeans consisting of 43% genistein, 21% daidzein and 2% glycetein (71, 72)] treated cells contained several γH2AX foci as detected 24 hours after the start of the treatment (p < 0.001). After 48 hrs treatment with SIFs, a more than twofold increase in the number of γH2AX foci and a greater frequency of cells positive for γH2AX foci (60-70%) was observed when compared with the 24 hrs time point (p < 0.001) (72). However, it would have been helpful for accurate data interpretation to take into the account the accumulation of cells in G2/M phase of the cell cycle, as the G2/M cells are characterized by increased numbers of γH2AX foci (73). Consistent with DNA damaging properties as determined with γH2AX detection, clastogenic activity (induction of micronuclei) was demonstrated for genistein at concentrations of 25 µM and above (69).

Catalytic topoisomerase II inhibitor activity, rather than topoisomerase II poison activity, was ascribed to daidzein (67). Consistent with properties of a non-inducer of DNA damage, daidzein has not caused any significant clastogenicity in NIH 3T3 cells (67). Analogously to daidzein, equol was found to be much less efficient in induction of DNA strand breakage than genistein (74). A study published by Constantinou and Husband, 2002 (75) have observed inhibition of the catalytic activity of topoisomerase II and stabilization of the topoisomerase II-mediated cleavable complex by idronoxil in a plasmid based cell free assay, indicating its activity as topoisomerase II poison. However, no cell culture studies have been published so far to confirm the findings of idronoxil activity on topoisomerase II in a cell system. In addition, idronoxil exhibits a significant structural similarity to equol (**Figure 1**), which has not been ascribed topoisomerase inhibitor properties to. Therefore, additional studies are warranted to clarify the potential activity of idronoxil as topoisomerase II poison.

### ENOX2 and PMET inhibition by idronoxil

Multiple studies have established a role of idronoxil as an inhibitor of plasma electron transport (PMET) (76–78). Idronoxil has been described as a potent inhibitor of a cancer associated splice variant of ENOX2 – tNOX, oncofetal protein (76), which is re-expressed by cancer cells upon transformation (79, 80). ENOX is a terminal oxidase of the plasma electron transport chain from cytosolic NAD(P)H *via* hydroquinons to acceptors at the cell surface (76). Idronoxil has been also demonstrated to inhibit PMET in tumor cells and primary immune cells (77, 78). However, its PMET inhibitory activity required much higher concentrations (IC50 of 46 µM in proliferating T cells), compared to that required for promotion of apoptosis (5.4 µM) (79). Therefore, it is more likely that idronoxil-induced apoptosis results from ENOX2 targeting, rather than PMET inhibition. More experimental evidence is needed to describe idronoxil effects on ENOX2 and PMET in more detail.

### Effects of isoflavones on the cell cycle

For genistein, induction of cell cycle arrest in the G2/M phase was observed, which is consistent with its properties as a potent inhibitor of both topoisomerases I and II (68). The G2/M cell cycle block occurred in a p53-dependent manner and was found to be associated with activation of ATM/P53, p21 and Gadd45α (81). Accumulation of cells in the G2/M phase is favorable for enhancing radiosensitization, as G2/M cells are characterized by increased radiosensitivity (82). Both equol and daidzein have been shown to induce an accumulation of prostate cancer cells in the G0/G1 phase of the cell cycle, the most radioresistant cell cycle phase (83). Furthermore, similar patterns of cell cycle responses were observed for idronoxil. Its administration leads to G0/G1 arrest (49, 84, 85) *via* p53-independent induction of p21WAF1/CIP1 (49). The potential of idronoxil to induce a G0/G1 block is consistent with its properties as ENOX2/PMET inhibitor as well as catalytic topoisomerase II inhibitor. ENOX2 proteins were ascribed an important role in driving cell enlargement (86). When idronoxil-treated cells fail to reach the size sufficient to pass the G1 checkpoint, they fail to divide, and as a result accumulate in the G0/G1 phase of the cell cycle.

### Inhibition of tyrosine kinases

Earlier studies (87) in extracellular systems and in cultured A431 cells have demonstrated an activity of genistein as a specific and potent inhibitor of tyrosine protein kinases, such as EGF. Interestingly, the study of genistein effects on serum-, E2-, and EGF-stimulated cell growth has revealed that EGF-R was not targeted by genistein at cytostatic concentrations in breast and prostate cancer cells, and only cytotoxic concentrations (more than 50 µg/ml) achieved a noticeable inhibition of EGF-R tyrosine phosphorylation (88, 89). Those results might indicate that drug is being metabolized or converted upon cellular entry, which leads to reduction of its intracellular concentrations. It also may selectively inhibit single sites of tyrosine phosphorylation of the EGF-R or engage with other signal transduction mechanisms below assay detection limit. It was also noticed that the daidzein is a much weaker inhibitor of serum-, E2- and EGF-stimulated cell growth.

The structure-activity relationship studies have revealed that removal of a hydroxyl group from the 5’ position as in flavone and daidzein (**Figure 1**) has drastically decreased their inhibitory activity on tyrosine kinase as compared to genistein (IC50 = 0.7 µg/ml in case of genistein, and >100 µg/ml for daidzein). Furthermore, the findings of tyrosine kinase inhibitory activity were not correlated with IC50 of growth inhibition of RSV-transformed 3Y1 (RSV3Y1) cells (7 µg/ml versus 25 µg/ml), providing additional evidence in support of different mechanisms of action of these compounds (90).

AKT is an important target and prognostic marker in prostate cancer and signification overexpression of AKT1, AKT2 and mTOR genes was observed in human prostate cancer samples compared with normal prostate gland tissue (91, 92). Genistein-mediated inhibition of AKT kinase activity, as well as inhibition of NF-kB activation was observed by Li and Sarkar, 2002 (26). However, the inhibitory effect on AKT and NF-kB was observed after the treatment with genistein for more than 24 hrs, and might simply result from genistein acting on targets upstream of AKT, or accompany the induction of cell death, therefore having less functional significance.

Furthermore, contradicting findings were reported in regards to the effects of idronoxil on AKT. One report has noticed its inhibition of AKT *via* dephosphorylation in renal cancer cells (50). However, in another study, an increase in phospho-AKT was detected after 6-12 hrs of idronoxil treatment. It was followed by rapid decrease just before the onset of apoptosis, which is not functionally significant (93). Moreover, activation of the Akt/FOXO3a pathway with another isoflavone, equol S-isoform has been implicated in inhibition of prostate cancer growth *in vitro* and *in vivo* (94).

### Isoflavone effects of isoflavones on cell adhesion and tumor metastatic potential

It has been demonstrated in rodent models and *in vitro* studies that genstein, administered at physiologically relevant concentrations of 10 nM inhibits cell detachment, protease production, cell invasion *via* inhibition of MMP-2 production (95–100) and human PCa metastasis (101) as well as B16BL6 murine melanoma pulmonary metastasis (102). Inhibition of FAK activation which regulates focal adhesion complex formation and turnover, has been demonstrated by several studies (103–105). Migration of rat prostate cancer cells, murine breast cancer and melanoma cells was found to be inhibited by genistein in a dose–dependent manner over the range of nontoxic concentrations of 1 to 10 µM (106, 107) possibly *via* inhibition of FAK phosphorylation (108).

### Cytostatic and cytotoxic activity of isoflavones

The activity of genistein in tumor cells has been characterized as being reversible and cytostatic at the lower concentrations of up to IC50 (89, 109) and becoming more cytotoxic with increase in concentration (89, 110). Another isoflavone, daidzein was found to be less effective than genistein at causing cell death. Up to 100 µM of daidzein was required to cause cytotoxicity comparable to that induced by 30 µM genistein as was determined in PC-3 cells (111).

In prostate and ovarian cancer cells reduction of cell proliferation caused by idronoxil was accompanied by induction of cell death (112, 113). It has been determined that idronoxil is at least five to thirty times more potent than genistein at decreasing proliferation of tumor cells (48, 49, 112). Furthermore, it has been established as a potent inducer of intrinsic and extrinsic apoptotic pathways operating in a caspase-independent fashion (113, 114). The evidence available so far has connected idronoxil to early effectors of cellular responses to stress, namely sphingolipid metabolism (115) and the PI3K/AKT pathway (112). The apoptotic effect appears to be linked to an increase in ubiquinol occurring as a result of ENOX2 inhibition, which in turn leads to cytosolic accumulation of NADH and decoupling of the sphingosine-1-phosphate (S1P) pro-survival signal transduction cascade (115, 116). It is accompanied by accumulation of ceramide through NADH-mediated activation of plasma membrane sphingomylelinase (117). Both events (concurrent reduction in S1P and accumulation of ceramide) appear to initiate cell death. Both genistein and idronoxil have been determined to exert antitumor activity in a p53-independent fashion as has been demonstrated in lung, prostate, and breast cancer cells (44, 48, 118).

Idronoxil potential as autophagy inhibitor has been demonstrated in ovarian clear carcinoma KK cells (117). Treatment with 0.5, 1 and 2 µg of idronoxil for 24 hrs caused inhibition of expression of the Atg7, Atg12 and Beclin 1 autophagy proteins, which was especially noticeable at 2 µg of idronoxil, the highest administered concentration.

The final product of daidzein metabolism, equol, has also shown very promising anticancer effects (45) and found to be about ten times more potent than daidzein (83).

### Selective targeting of tumor cells by isoflavones

Selectivity of isoflavones towards tumor cells was observed for several cell types. Genistein has not caused any significant induction of apoptosis in spontaneously immortalized ‘normal’ MCF10A and MCF12A breast epithelial cells *in vitro* as assessed after 72 hrs of drug exposure. On the contrary, apoptosis has been strongly induced in a p21-dependent fashion by 90 µM of genistein in malignant MDA-MB-231 and MCF10CA1a breast cancer cells (118). However, this study results are inconclusive as apoptosis was measured only at one time point of 72 hrs. This leaves the possibility open that induction of apoptosis in immortalized breast epithelial cells has occurred at a later time, or cells would undergo proliferative cell death leading to a degree of clonogenicity loss equal to that of malignant cells (118). The authors have also addressed the effects of genistein on the cell cycle. Pronounced induction of G2/M arrest by genistein was observed in all tested cell lines. The same group has also demonstrated that CRL-2221 prostate cells transformed with human papilloma virus 18 (HPV-18) were less sensitive to genistein than PC-3 prostate tumor cells (27). In comparison, the earlier study (83), which was performed in true primary prostate epithelium cell cultures, has observed the opposite effect. A range of non-transformed prostate epithelial cells was shown to be even more sensitive to both genistein and equol than tumor cells at day 6 of continuous incubation with concentrations attained as a result of dietary soy uptake in Asian men (83).

Genistein effects on proliferation and toxicity was also assessed in normal and tumor blood cells (109, 110). Normal proliferating lymphocytes were shown to be resistant to 24 hrs exposure to genistein at concentrations as high as 200 µg/ml, whereas 50% inhibition concentration after 24 h of exposure for HL-60 and MOLT-4 cells was 8.5 and 13.0 µg/ml, respectively (109). Leukaemic CFU-AML cells were much more sensitive to genistein than normal CFU-GM from either marrow, unseparated blood, or CD34+CD45RA– cells derived from blood (110).

Several studies have provided consistent indications of enhanced sensitivity of tumor cells to idronoxil. In comparison, wild-type mouse embryonic fibroblasts were found to be resistant to idronoxil (117). It was also noticed that the potential of idronoxil to inhibit proliferation and cause apoptosis in blood cells was to some extent correlated to the proliferation activity of the studied cells (78). The isoflavone reduced proliferation and caused cell death in primary stimulated lymphocytes, however the resting lymphocytes were more resistant. Idronoxil was also found to be more cytotoxic in acute lymphocytic leukaemia cells and cell lines, than in activated T cells, where acute myeloid leukaemia cells demonstrated a wide range of sensitivities to it (78). The authors hypothesized, that this effect was related to a idronoxil activity as a PMET inhibitor, as rapidly proliferating cells are known to have high demand in energy. Idronoxil was also found to exert antiangiogenic antiproliferative effects on normal endothelial cells, and no significant toxicity was observed on unstimulated endothelial cells (119). However, it could as well result from its hypothesized activity as topoisomerase II inhibitor in cells not expressing ENOX2. This is supported by the available evidence, which demonstrate that resting T- and B- lymphocytes are resistant to activity of topoisomerase inhibitors etoposide and camptothecin, and acquire sensitivity during transition from G1 to S phase of the cell cycle (120).

### Concentration dependent effects

As isoflavones undergo extensive metabolism and bioconjugation, it is important to consider that the concentration of free isoflavones such as genistein is in the low nanomolar range in the blood of soy consumers. Therefore, effects induced by low concentrations potentially represent important clinically relevant mechanisms. Relevance of effects low-to-mid micromolar range is less clear as only total genistein (conjugated plus free forms) can reach micromolar concentrations in the blood after high-dose genistein administration.

Concentration-dependent biphasic activity was described for both genistein and idronoxil across different cell types. At a lower concentration range genistein was ascribed an ability to stimulate estrogen-dependent human breast tumor growth (121, 122). An ER-dependent biphasic effect was observed for genistein in ER-β expressing MCF7 cells. Increase in cell proliferation was noted at 0.1–10 µM of genistein, whereas concentrations of 20–90 µM have led to suppression of cell proliferation and DNA synthesis (123). Similarly in CHO-K1 cells characterized by high expression of ER-beta over ER-alpha, single treatment of genistein at physiologically achievable low concentration < 2 µM induced proliferation, while high concentrations (50 and 100 µM) resulted in suppression of cell proliferation, G2/M arrest and induction of apoptosis consistent with biphasic estrogenic, anti-estrogenic and DNA topoisomerase II targeting effects of genistein (124). Furthermore, lower concentrations of genistein appeared to stimulate some immune effects, however it acted as immunosuppressant at higher concentrations. When administered at non-physiologic high concentrations (10–100 mmol/L), genistein was found to inhibit cytotoxic T-cell–mediated tumoricidal activity, alter leukocyte adherence, impair T-cell motility, and inhibit the activation of natural killer (NK) cells in response to lipopolysaccharide or fixed bacteria [reviewed in (125)].

Idronoxil at low concentrations (0.05-0.5 µg/ml) enhanced the *in vitro* cytotoxic responses of PBMC (peripheral blood mononuclear cells) and was demonstrated to specifically activate by several fold the killing capacity of NK cells (126). However, at concentrations >1 µg/ml (4 µM) it was found to inhibit proliferation and reduced the viability of healthy donor-derived PBMC (126).

At high concentrations idronoxil was found to stimulate apoptosis in normal trophoblasts (127). Idronoxil alone had no effect on neurite outgrowth and length in a concentration range up to 1 µM, however administration of 10 µM idronoxil resulted in neurite toxicity (128). The concentrations of 30-200 µM of genistein were reported to be efficient at induction of apoptotic cell death in a range of cancer cell lines (27, 43, 46, 47, 71, 129–132).

It is likely that the observed biphasic mode of the isoflavone effects on various cell types, including immune cells, results from the concentration-dependent target(s) activation. For example, effects evoked by lower concentrations of genistein result from its engagement of ER receptor associated pathways, whereas high concentrations achieve topoisomerase II targeting, hence growth inhibition, effects on cell cycle and anti-proliferative action can be observed.

### Immune cell activation as antitumor mechanism of isoflavones

It could be hypothesized that most of the systemic and clinically significant antitumor activities of isoflavones might involve activation of immune cells. Tumor targeting effects of phytoestrogens might be explained not only by direct activity towards tumor cells (36, 88, 133), but also by their indirect action *via* activation of the immune system. As a result of genistein administration, increased host resistance to B16F10 tumor cells has been observed in mice and was accompanied by dose related increase of cytotoxic T-cell activity and interleukin (IL)-2-stimulated natural killer (NK) cell activity (134).

Both genistein and dadzein at nutritionally relevant concentrations, genistein at <0.5 µM/L and genistein and dadzein glucuronides at 0.1-10 µM/L, were found to enhance human NK cell toxicity *in vitro* (135). Consistent with biphasic dose response, genistein at concentrations higher than > 0.5 µM/L has significantly inhibited NK cytotoxicity (P < 0.05).

In several studies reviewed by Sakai and Kogiso, 2008 (136) genistein has been shown to enhance the cytotoxic response mediated by NK and cytotoxic T-cells, and T-cell cytokine production. Furthermore, chemopreventative properties of genistein were shown to be associated with induction of antitumor immunity in adult female mice (137). At the same time it was found to suppress antigen-specific immune response *in vivo* and lymphocyte proliferation response *in vitro* (136).

Georgaki et al, 2009 (126) has demonstrated that idronoxil is able to enhance the *in vitro* cytotoxic responses of PBMCs *via* activation of the killing capacity of NK cells by several fold. It reduced the tumor burden and prolonged survival in a mouse model of colon cancer (126).

The mechanisms of the observed isoflavone effects on the immune cells are unclear. Isoflavones might exhibit their antitumor activity indirectly, activating immune cells *via* ER. Immune cells are known to express sex hormone receptors including ERα and ERβ, which has been shown to underlie sex differences in immune cell numbers and/or functional responses of the innate and the adaptive immune systems (138–140). Estrogen and ERα signaling has been shown to promote type I IFN synthesis (141–144), and influence levels of proinflammatory cytokines (IL-12, IL-6, IL-1b), and IL-10 (145). Activation of ER receptor mediated immune pathways might improve the antitumor responses.

Another important potential mechanism of engaging beneficial antitumor immune responses includes idronoxil alteration of pyridine nucleotide products of plasma membrane redox, NAD+ and NADH, which stimulate sphingomyelinase (SM) and inhibit sphingosine kinase (SK1) which in turn leads to loss of sphingosine-1-phosphate (S1P) (93). Increased SK1 expression has been observed in several tumor types, and in some cases has been correlated with disease progression and reduced patient survival (146). Furthermore, S1P and its receptors play a role in migration of immune cells (147, 148) and SK1 has been recently shown to act as a key regulator of antitumor immunity (149). SK1 silencing decreased the expression of immunosuppressive factors in the tumor microenvironment which normally limit infiltration with regulatory T cells. Interestingly, the recent study has demonstrated a potential of autophagy inhibition to promote antitumor immunity, which in turn mediated an improved control of distant non-irradiated lesions *via* systemic type I IFN signaling (150). Further studies should establish a potential relevance of autophagy inhibiting activity of idronoxil (117) to its antitumor efficacy when combined with radiation therapy.

## Combination effects

Radiation therapy achieves the best success rate in combination with neoadjuvant or adjuvant chemoradiotherapy. The exploration of anticancer activities and mechanisms of cancer prevention by isoflavones has provided evidence of their potential to enhance anticancer therapies.

The ability to modify the radiation response in tumors and normal tissues by a mixture of soy isoflavones and genistein has been observed and explored by several research groups, whereas no radiosensitizing effect was observed with another isoflavone, daidzein (151).

The activity of idronoxil in combination with cancer therapy focusing on its chemosensitizer properties (152–156) and its ability to overcome chemoresistance to gemcitabine and doxorubicin and sensitize to 5-FU and oxaliplatin-induced apoptosis (156) was extensively studied in several tumor types including ovarian and osteosarcoma cancer cells. Furthermore, current clinical trials of Veyonda, the suppository formulation of Idronoxil (current name for idronoxil) in combination with external or targeted radiation hold promise to provide a novel potent therapy of end-stage prostate cancer (**Table 1**).

**Table 1:** Completed and ongoing clinical trials of isoflavones in combination with radiation therapy.

Study Name (NCT Number/UTN)	Phase	Patient Population	Study desiga	Type of radiation therapy	Primary Endpoint	Secondary Endpoint
Phase II Randomized Study of Soy Isoflavones in Patients With Localized Prostate Cancer Treated With Radiation Therapy (NCT00243048)	Phase II	70 patients with prostate adenocarcinoma receiving curative RT	Oral soy protein isolate (isoflavones) twice daily for 6 months in the absence of disease progression or unacceptable toxicity.	External	Oxidative DNA damage at 3 and 6 months	Quality of life at 3 and 6 months; toxicity as measured by number and grade of adverse events weekly during radiation and at 3 and 6 months
A Phase II Trial of Phenoxodiol in Patients With Castrate and Non-Castrate Prostate Cancer NCT00557037	Phase II	60 patients Grp A: Patients with chemotherapy naïve androgen independent disease Grp B: Patients with rising PSA after radical prostatectomy or radiotherapy that are androgen	Phenoxodiol in oral capsule, 400 mg every 8 hours daily, for 12 weeks - assement to a maxinum of 12 months	Post external radiation therapy (Grp B)	The proportion of patients that have a 50% post-therapy PSA decline at 12 weeks in patients	The proportion of patients treated with phenoxodiol that have measurable disease regression at 12 weeks
**Study Name (NCT Number/UTN)**	**Phase/ status**	**Patient Population**	**Study design**	**Type of radiation therapy**	**Primary Endpoint**	**Secondary Endpoint**
Idronoxil suppository Combined with Radiotherapy for Metastatic Prostate Cancer (NCT03041285)	Phase l/active, recruiting	12 castrate- resistant metastatic prostate cancer with 2-3 lesions suitable for radiotherapy	Dose escalation (sequential assignment)	External (Stereotactic) Radiation Therapy	Safety of Idronoxil dose escalation	Evidence of clinical tumor response
NOX66 and Palliative Radiotherapy in Patients With Late-Stage Prostate Cancer - a Phase 1b Proof of Concept and Dose Confirmation Study	Phase l/active, not recruiting	26 participants	Dose escalation (sequential assignment)	External radiation therapy	Change of incidence of Treatment- Emergent Adverse Events including SAEs	Change of tumour size; change of non-target lesions; overall response
Radionuclide therapy using 177Lu-PSMA: extension of a pilot study in men with castrate- resistant prostate cancer to determine the clinical benefit of combination therapy with idronoxil (UTN U1111-1206- 1132)	Phase I/ll/active, recruiting	56 males with metastatic castrate resistant cancer of the prostate	Dose escalation (24 patients - -1200 mg; The 400 mg daily cohort will be compared to the 800 mg daily cohort.	Lul77-PSMA targeted therapy	To determine the toxicity profile of combination therapy of idronoxil and 177Lu-PSMA	To document the anti- cancer efficacy using 177Lu-PSMA-idronoxil combination therapy using a composite of surrogate measures including serum PSA, medical imaging, EORTC QLQ-C30 and
A Pilot Study of Soy Isoflavone, Genistein, in Combination With Radiation Therapy and Cisplatin in Locally Advanced Squamous Cell Carcinoma of the Head and Neck (NCT02075112)	Phase l/active, not recruiting	25 patients with squamous cell carcinoma of the head and neck (SCCHN); Clinical stage III or IV	70 Gy in 2 Gy/day combined with Cisplatin 100 mg/m² on days 1, 22, and 43 of RT, and genistein 150 mg daily for the duration of RT	External radiation therapy	Percentage of patients with grade 2 or higher xerostomia at 1 year post-treatment	Biomarker studies: changes in interleukin 6; vascular endothelial growth factor, vascular endothelial growth factor

### Potentiation of radiation toxicity in *in vitro* studies

Most of the studies have focused on sensitization of cancer cells to radiation with genistein, whereas not much is known about interaction of other isoflavones with irradiation. Pre-treatment with genistein prior to irradiation resulted in radiosensitivity enhancement in tested PC-3 (prostate), Me180 and CaSki (cervical cancer), BR-231, MCF-7, MDA-MB-231 (breast), as well as KCI18 and RC-2 (renal cell carcinoma) and sarcoma cancer cell lines (151, 157, 158). In PC-3 cells genistein appeared to inhibit radiation-induced NF-kB activation and promoted apoptosis as detected by PARP cleavage (158).

### Optimization of the drug administration schedule

The timing of drug administration can be crucial for achieving maximal radiosensitization (159). It has been observed that administration of genistein 24 hours prior to irradiation and its presence in the cell culture medium during the exposure resulted in a better radiosensitization effect than when the drug was administered post-irradiation (151, 158). Furthermore, continuous exposure of cells to genistein was found to be required for maximal effect, as cell growth inhibition induced by genistein in combination with radiation was dependent on its presence in the medium during the 10-day incubation of the colony assay (7). Interestingly, a significant synergistic effect and radiosensitization was observed in H35 hepatoma cells with 30-90 µM of genistein administered only 30 min prior to irradiation, and present in cell culture medium for further 24 hrs post-IR. No effect of genistein on survival of irradiated cells was found when they were pre-incubated for periods of up to 24 hrs (160).

Potentiation of the radiation effect with genistein in cervical cancer cells Me180 and CaSki was observed with administration of 10 µM of genistein at 48 hrs prior to irradiation (151). Radiation enhancement ratios, defined as survival with irradiation and genistein compared to radiation alone, were found to vary from 1.4 to 4.4 over the range of doses. 10 µM of genistein was found to be mildly cytotoxic in cell growth assays. Induction of G2/M arrest by genistein was observed only in Me180 cells, and some accumulation of cells in S phase was observed in CaSki cells. The authors found significant inhibition of Mcl-1 by genistein which correlated with an increase in radiosensitivity in Me180 cells. Inhibition of activated pAKT (Thr 308) with genistein and radiation was noticed in CaSki cells. Using the dosage of 10 μM genistein, the sensitizer enhancement ratios after exposure to X-rays at a 10% cell survival (IC10) were established to 1.43 for MCF-7 and 1.36 for MDA-MB-231 cells, respectively (157, 158).

A significant potentiation of radiation cytotoxicity by genistein was observed in HL-60 leukaemia cells (161). Exposure to 5 Gy alone has caused an increase in sub-G0/G1 population up to 21.2% indicating cell death at 48 hrs post-irradiation. Pre-treatment with 20 µM of genistein alone resulted in 27.5%, and combination with irradiation has led to accumulation of about 60% cells in sub-G0/G1 cell population. The authors have also found that genistein exerted a somewhat radioprotective effect on normal lymphocytes (161). In another study (162) a significant protection of B-lymphocyte precursors by genistein was observed after administration at 30 µg/ml 30 min prior to irradiation and continuous incubation for 24 hrs.

### Effects of isoflavones on repair of radiation-induced DNA damage

A study published by Singh-Gupta et al., 2011 (72) demonstrated that SIF potentiated DNA damage induction by irradiation in human A549 non-small cell lung cancer cells. Pre-incubation with SIF for 24 hrs prior to exposure to 3 Gy has resulted in induction of 21.15 ± 2.38 of γH2AX foci/cell, which is almost double of foci induced by IR alone at 1 hr post-exposure (13.86 ± 2.31 of γH2AX foci per cell), or four times of foci resulted from incubation of cells with SIF for 24 hrs (4.96 ± 1.02 foci per cell). The effects of SIF on the functioning in the base excision repair pathway have also been investigated in this study. The authors suggest that the observed potentiation of residual DNA damage with SIF occurs *via* inhibition of the repair activity of apurinic/apyrimidinic endonuclease 1/redox factor-1 (APE1/Ref-1), the main player in the cellular response to DNA damage and redox regulation against oxidative stress, by analogue with methoxyamine (72). The mode of interaction between isoflavones and radiation cannot be reliably determined as 3 Gy of irradiation administered alone resulted in 80% reduction in survival, respectively (72).

Study by Liu et al, 2013 (157) demonstrated that pre-treatment of MCF-7 (ER-positive, harboring wild-type p53) and MDA-MB-231 (ER-negative, harboring mutant p53) cells with genistein for 24 hrs followed by 4 Gy of X-rays resulted in increased number of the γ-H2AX foci at 12 h post-irradiation and decreased homologous recombination repair protein Rad51 foci formation. Genistein has significantly potentiated accumulation of cells in radiosensitive G2/M phase of the cell cycle phase and apoptosis.

Taken together, the published results of cell culture experiments and pre-clinical models indicate the potential of soy isoflavones to modify the radiation response of tumor cells. Ideally, the radiosensitization is defined as increased radiation sensitivity of cells in the absence of significant drug-induced cytotoxicity. In the case of genistein, most published studies show describe enhancement of radiation effects by the soy isoflavone administered at weakly cytotoxic concentrations in the range of 5–15 µM. It should also be noted that concentrations of genistein in this range are considered mild and being close to physiological doses achieved as a result of dietary supplementation (14, 163).

### Effects of isoflavones on radiation-induced invasion and migration

Recent publication by Liu et al, 2021 (164) has demonstrated the potential of genistein to inhibit radiation-induced invasion and migration of DNA-PKcs-positive glioblastoma cells. The authors have observed interaction of genistein with DNA-PKcs which resulted in blocking DNA-PKcs/Akt2/Rac1 signaling pathway induced by radiation in DNA-PKcs-positive but not in negative glioblastoma cells and established the exact binding site of genistein to DNA-PKcs. The physiological relevance of these findings remains to be further addressed.

### Preclinical models of radiosensitization with isoflavones

Several research groups have explored the aspects of interaction of genistein and soy products with irradiation in pre-clinical mouse models. Potentiation of radiation inhibition of tumor growth by genistein has been observed in the orthotopic nude mouse model of prostate cancer (165). When 5 mg/day of genistein was administered orally for 2 days prior to exposure to 5 Gy, and every other day for 4 weeks post-irradiation, it led to greater inhibition of primary tumor growth (87%) compared with genistein (30%) or irradiation (73%) alone, and improved survival. No signs of any toxicity which potentially could be associated with genistein treatment were observed (165). Intriguingly, when genistein was used as a single treatment, it promoted metastasis to regional para-aortic lymph nodes. This metastasis-promoting activity was also observed in a mouse model of renal cancer (166). Treatment of established kidney tumors with genistein demonstrated a tendency to stimulate the growth of the primary kidney tumor and increase the incidence of metastasis. However, when given in conjunction with radiation therapy, genistein significantly inhibited the growth and progression of established kidney tumors (166). These results confirm the potentiation of radiotherapy by genistein. Raffoul et al., 2007 have demonstrated that administration of soy isoflavones in the nude mouse model of prostate cancer with implanted PC-3 cells mediated reduction of the prostate weight from 420 mg to 250 mg, and in combination with irradiation from 100 mg to 50 mg (167). In contrast to the studies where administration of pure genistein promoted metastases (165, 166), no such pro-metastatic effect was observed with combination of soy isoflavones. Subsequently, it has been determined in the PC-3 prostate tumor model that dadzein is a compound in the SIF mixture capable of negating genistein-induced metastasis (111). Another study confirmed that daidzein and its derivative equol inhibit the cancer cell invasion in breast cancer cells potentially *via* down-regulation of matrix metalloproteinase-2, a major matrix degrading enzyme (168). Interestingly, the angiogenic properties of idronoxil were demonstrated, too and occurred *via* inhibition of endothelial cell proliferation, migration and capillary tube formation as well as inhibition of expression of the matrix metalloproteinase MMP-2 (118).

### Radioprotective properties of isoflavones explored in pre-clinical models

Numerous studies have described the radioprotective effect of genistein in mouse models of radiation-induced toxicity.

In male mice genistein mitigated radiation-induced testicular dysfunction by an anti-apoptotic effect and recovery of spermatogenesis (169). Induction of IL-6 and serum granulocyte-colony stimulating factor (170, 171) and increased hematopoiesis (172) was found to be associated with genistein-mediated protection of irradiated mice. Davis et al., 2007 have shown a significant radioprotection of the hematopoietic progenitor cell compartment by a single subcutaneous administration of genistein 24 hrs prior to irradiation *via* induction of quiescence in hematopoietic stem cells (173, 174).

Another study published by Day, et al. (175) has also demonstrated significant radioprotective properties of genistein in normal tissues. 200 mg/kg genistein delivered subcutaneously protected from acute radiation injury in mice after total body exposure to 7.75 Gy. The authors observed that at 30 days genistein has led to improved survival from 23-53% to 92%, accompanied by reduced lung damage. Mitigation of the radiation induced lung injury for a period of up to 28 weeks was demonstrated by Mahmood et al., 2011 (176). A study led by Hillman (177) has further explored the exact mechanisms of radioprotective effects of genistein in normal tissues. It has been established that pre-treatment of mice with SIF for three days prior to lung irradiation, and continuous treatment at various doses for 18 weeks post-exposure resulted in reduction in infiltration and activation of alveolar macrophages and neutrophils in both the bronchoalveolar and lung parenchyma compartments. Soy treatment protected the F4/80+CD11c- immunoregulatory interstitial macrophages, which are normally decreased by irradiation, and led to switch from a pro-inflammatory M1 macrophage to an anti-inflammatory M2 macrophage phenotype (177).

Using pre-clinical mouse cancer models, studies on the combination of soy isoflavones with tumor irradiation demonstrate a significant anti-cancer effect by these two modalities and indicate the potential and safety of dietary factors to improve conventional radiotherapy for a better control of tumor.

## Clinical trials

Combination of isoflavones with radiation therapy yielded good indications of its efficacy and safety in clinical trial in localized prostate cancer (178), the most common cancer in men (179). Despite high level of diagnosis, the relatively successful treatment of localized disease with surgery or radiotherapy results in the 5-year overall survival rate above 90% (179). However, treatment morbidity leads to worsened quality of life. The results of the pilot clinical study which was published in 2010 by Ahmad et al. (178), suggested that soy isoflavones taken in conjunction with curative radiation therapy could reduce the urinary, intestinal, and sexual adverse effects in patients with localized prostate cancer. 200 mg soy isoflavone or placebo were administered orally daily in a group of 42 patients for 6 months beginning with the first day of radiation therapy, which was given in 1.8 to 2.5 Gy fractions for a total of 73.8 to 77.5 Gy. At 3 months, soy-treated patients had less urinary incontinence, less urgency and better erectile function compared to the placebo group, and at later time point of 6 months the symptoms in the soy-treated group had further improved. Patients experienced less dripping/leakage of urine (7.7% in Group 1 vs. 28.4% in Group 2), less rectal cramping/diarrhea (7.7% vs. 21.4%), and less pain with bowel movements (0% vs. 14.8%) than placebo-treated patients. Another clinical study in pediatric cancer patients has demonstrated that genistein reduced the adverse effects of abdominal radiotherapy. Patients experienced less pain and no diarrhea, which are common side effects of abdominal radiation (180).

However, there is limited clinical data available on isoflavone antitumor efficacy when combined with radiation therapy (**Table 1**). Several clinical trials have investigated the efficiency of idronoxil in combination with irradiation in late stage metastatic chemotherapy resistant prostate cancer (mCRPC) patients and in head and neck cancer patients (**Table 1**) (181–185). PoC and dose confirmation DARRT study in late-stage prostate cancer patients has addressed safety and potential indications of efficacy of the combination of 1 of 3 doses of idronoxil, given as suppository (NOX66) in dose-escalated fashion (400mg, 800mg and 1200mg) in combination with 20Gy of EBRT given over 5 daily fractions to selected target lesion/s for all cohorts.

The combination of idronoxil with a novel promising treatment for late-stage prostate cancer [^177^Lu]Lu-PSMA-targeted radionuclide therapy with is also being explored as part of the LuPIN clinical trial (182–185). Recently published results (184, 185) of phase I/II study in 56 men with progressive mCRPC with extensive prior treatment which included docetaxel and cabazitaxel in addition to abitraterone and/or enzalutamide indicated safety and tolerability of NOX66 (Idronoxil) delivered at doses up to 1200 mg in suppository form. Suppositories were given on days 1-10 of a 6-week cycle, where 7.5 GBq of [^177^Lu]Lu-PSMA-617 was delivered on day 1 of a 6-week cycle. 86% of patients have experienced a >50% PSA decline, which was higher than reported in prospective study of [^177^Lu]Lu-PSMA-617 (56% of PSA decline) (186–188), and the randomized TheraP trial (66%) (185, 190). Pain reduction was observed in more than half of the men. The median overall survival (OS) was found to be 19.7 months (95% CI 9.5– 30 mo), whereas previously reported median OS in study of [^177^Lu]Lu-PSMA-617 efficacy in mCRPC was found to be 13.3 months (95% CI 10.5-18.7) (189). These results indicate a potential of NOX66 to improve survival outcome in mCRPC patients. Further research is required to better evaluate the efficacy of NOX66 and LuPSMA-617 combination.

## Conclusions

Unique property of soy isoflavones specifically genistein to target tumor cells while sparing normal tissues when combined with radiation have been addressed in a number studies. Showing some structural resemblance, yet causing significantly different biological effects, other isoflavones such as idronoxil are currently being successfully explored to clinical benefit in combination therapies.

Identification and characterization of isoflavone targets has been a continuous endeavor spanning over decades. Activity of isoflavones as protein kinase inhibitors and DNA topoisomerase II inhibitors/poisons has been extensively investigated. However, it might have limited clinical implications as it occurs at supraphysiological concentrations, which are manyfold greater than concentrations that can be obtained through consumption of dietary genistein or even through the use of supplements containing genistein. Limited available evidence do not support kinases as direct targets of idronoxil, and not much is known in regards to its activity on topoisomerases. This leaves ER and ENOX2/PMET as well described potential molecular targets mediating cellular effects of idronoxil in tumor and normal cells. However the activity of idronoxil towards other targets cannot be excluded.

The S1P inhibition properties resulting from ENOX2/PMET targeting in combination with potent apoptosis inducer characteristics of idronoxil hold great promise in indirectly potentiating antitumor therapies *via* engagement with immune cells. There is also an array of other biological actions of isoflavones including those on cell communications (191) and mitochondrial biogenesis, which need to be better understood.

Completed and ongoing clinical trials are set to explore the clinical utility of phenoxodiol in combination with different forms of radiation therapy, especially targeting patients with unmet need for treatment options. Combination of soy isoflavones with radiotherapy appears to hold the most therapeutical promise, however, the precise mechanisms and molecular targets need to be better understood.

## Author contributions

The author confirms being the sole contributor of this work and has approved it for publication.
